# Learning from fungicide resistance: Evolutionary insights to guide RNAi-based control of fungal crop pathogens

**DOI:** 10.1016/j.fbr.2025.100443

**Published:** 2025-09

**Authors:** Joris A. Alkemade, Nichola J. Hawkins, Elena Baraldi, Alan G. Buddie, Helen M. Cockerton, Isabel Corkley, Bart A. Fraaije, Ester Gaya, Danna R. Gifford, Florian Hartig, Kostya Kanyuka, Aline Koch, Jonatan Niño Sánchez, Gail M. Preston, Michael F. Seidl, Pietro D. Spanu, Bernhard T. Werner, Joy Lyu, Timothy G. Barraclough

**Affiliations:** a Department of Biology, University of Oxford, Oxford, UK; b Calleva Centre, Magdalen College, Oxford, UK; c National Institute of Agricultural Botany (NIAB), Cambridge, UK; d Department of Agricultural and Food Sciences, University of Bolonga, Bologna, Italy; e CAB International (CABI), Ascot, UK; f School of Biosciences, University of Kent, Canterbury, UK; g Sustainable Agricultural Systems, RSK ADAS Ltd, Wolverhampton, UK; h Net Zero and Resilient Farming, Rothamsted Research, Harpenden, UK; i School of Agriculture, Policy and Development, University of Reading, Reading, UK; j Wageningen University & Research, Wageningen, the Netherlands; k Comparative Plant and Fungal Biology, Royal Botanic Gardens Kew, Richmond, UK; l School of Biological Sciences, Faculty of Biology Medicine & Health, The University of Manchester, Manchester, UK; m Theoretical Ecology, University of Regensburg, Regensburg, Germany; n Department of Plant Production and Forest Resources, Sustainable Forest Management Research Institute (iuFOR), College of Agricultural Engineering (ETSIIAA), University of Valladolid, Palencia, Spain; o Theoretical Biology & Bioinformatics, Utrecht University, Utrecht, the Netherlands; p Imperial College, London, UK; q Liebig University Giessen, Giessen, Germany

**Keywords:** RNAi, Crop protection, Fungicide resistance, Fungal pathogens, Evolutionary genomics

## Abstract

Crop protection against fungal pathogens is essential to prevent crop losses and maintain food security. Current crop protection relies heavily on chemical fungicides. However, rapid evolution of fungicide resistance, the constant appearance of new pathogens, and legislation against chemical pesticides due to concerns regarding their impact on human health and the environment, mean new crop protection strategies are urgently required. One elegant solution is double-stranded RNA-based crop protection, which aims to silence selected genes in the pathogen to reduce crop damage. This technology brings the promise of targeting specific genes, which could be chosen to maximise protection, minimize off-target effects and reduce the risk of resistance evolution. Here we discuss strategies for successful use of this novel technology based on lessons learned from fungicide resistance and recent discoveries in fungal evolution derived from genome-sequencing.

## 1 Introduction

Fungal pathogens account for the loss of an estimated 10–23% of global crop production annually, translating to economic losses in the billions of dollars and posing a significant threat to global food security and economic stability (FAOSTAT, 2021; Savary et al., 2019). To mitigate these losses and minimize agricultural land use expansion, resistance breeding and effective crop protection are essential (Balmford et al., 2018). Current crop protection strategies, however, are controversial as they depend heavily on the extensive use of fungicides in both conventional and organic farming (Beckerman et al., 2023). Conventional agriculture primarily employs synthetic chemical fungicides, whereas organic production systems often rely on copper salts, sulphur and mineral oils (Edwards-Jones and Howells, 2001; Gupta, 2018, 2022; Katsoulas et al., 2020; Tamm et al., 2022). This dependency on fungicides not only imposes substantial economic burdens but also poses significant risks to human health and the environment. Effective disease management is increasingly difficult due to shifting patterns of disease dispersal driven by global trade and climate change (Fones et al., 2020; Raza and Bebber, 2022), and is further complicated by the rapid evolution of pathogen populations, with resistance emerging across all classes of pesticides and antimicrobials (Fisher et al., 2018; Hawkins and Fraaije, 2018).

Current disease management strategies to mitigate resistance combine chemical and non-chemical control measures, such as the deployment of resistant crop varieties and crop sanitation. In agriculture, these principles are embodied in Integrated Pest Management (IPM; Dara (2019); Barzman et al. (2015)), an ecosystem-based approach emphasizing long-term prevention through practices such as deploying resistant crop varieties, biological control, and crop rotation, with pesticides used only as a last resort. However, in practice, chemical crop protection remains the dominant form of disease control and resistance evolution continues to undermine its effectiveness (Beckerman et al., 2023). Growing concerns about public health, environmental impact, and consumer and policy preferences for reduced agrochemical use have led to the banning of certain fungicides, adding further pressure on food supplies (Burandt et al., 2024; Hillocks, 2012). The cumulative pressures of pathogen evolution, emergence and range expansion due to climate change, and shifting consumer demands, highlight the limitations of conventional crop protection strategies. This has created an urgent need for innovative approaches to secure and enhance future food production sustainably.

One promising tool to improve crop protection is to use RNA interference (RNAi)-based crop protection to downregulate specific genes in a pathogen with the aim to suppress disease progression (Qiao et al., 2021; Rosa et al., 2022; Zhao et al., 2024). There are two main approaches for inducingRNAiin plants: spray-induced gene silencing (SIGS), which involves foliar application of double-stranded RNA (dsRNA), and host-induced gene silencing (HIGS), which relies on engineering the plant to produce dsRNA internally. In this work, we focus on SIGS, as it does not require genetic modification and offers greater flexibility for managing resistance due to faster development, fewer regulatory hurdles, and greater consumer acceptance (Cagliari et al., 2019; Spina et al., 2025). Nonetheless, considerations for selecting pathogen target genes to optimize effectivity and reduce resistance risk, will apply for both application methods.

The potential for high specificity of dsRNA and its short half-life in the environment offers a promising solution to the limitations of conventional fungicides (Rosa et al., 2022). Potentially, dsRNA methods may limit, delay or counter pathogen adaptation with lower risk of off-target effects than conventional fungicides (Zhao et al., 2024). However, any method that reduces pathogen fitness inevitably exerts selective pressure in favour of less susceptible variants. Consequently, lessons from past resistance evolution combined with a deep understanding of how pathogens evolve is crucial to deploy new methods in a durable way. This article explores lessons learned from the evolution of resistance to existing fungicides, together with recent discoveries in fungal evolution from genome-sequencing, to discuss the most promising strategies for dsRNA-based control and examine potential challenges associated with the successful implementation of this novel technology.

## 2 Lessons from chemical control

Fungicides have been the dominant method of controlling plant pathogens since the early 20th century (Beckerman et al., 2023). The emergence of resistance has been well documented by international consortia such as the Fungicide Resistance Action Committee (FRAC) and European and Mediterranean Plant Protection Organization (EPPO). The history of fungicide usage and the evolution of resistance provides useful insights for designing new control methods, which we discuss here focusing on the most relevant aspects for resistance management in relation to dsRNA strategies.

### 2.1 Fungicide history

The earliest chemical control methods relied on inorganic compounds like copper sulphate and sulphur and these are still used in organic agriculture despite their harmful properties (Burandt et al., 2024). Organic multi-site fungicides like dithiocarbamates, phthalimides and chloronitriles, which combined preventative activity with a low risk of resistance, were introduced in the mid-20th century (Morton and Staub, 2008). However, they lacked curative power or systemic mobility, and often have off-target toxic effects, which has led to recent bans of mancozeb and chlorothalonil in the EU and UK. The 1960s saw the introduction of site-specific fungicides, such as benzimidazoles, which targeted the β-tubulin protein, a key component of microtubules. These were highly effective, with systemic and curative activity, but the first cases of resistance to benzimidazoles were reported within a few years, spurring the development of newer fungicide classes (Lucas et al., 2015). Demethylation inhibitor (DMI) fungicides, such as the azoles introduced in the 1970s, targeting sterol biosynthesis for fungal membranes, represented another breakthrough. They offered systemic activity and curative potential, but their effectiveness has been eroded as pathogens developed mutations in the target cytochrome P450 enzyme CYP51 (sterol 14α-demethylase; Becher and Wirsel (2012)). By the 1990s, strobilurins emerged, targeting mitochondrial respiration via cytochrome *b*. Despite their innovative mechanisms, widespread resistance quickly emerged in many key pathogens (Fernández-Ortuño et al., 2008). Subsequent new classes such as succinate dehydrogenase inhibitors (SDHIs) and quinone-inside inhibitors (QiIs) offered highly targeted modes of action but SDHI resistance has now emerged in several pathogens (Sierotzki and Scalliet, 2013), and Qils are at high-risk for resistance emergence and require strategic use to prolong their effectiveness (Fouché et al., 2022).

### 2.2 Modes of resistance

Fungicide resistance mechanisms broadly fall into two main categories: target-site and non-target-site resistance (Dorigan et al., 2023). Target-site resistance includes mutations or overexpression of target genes, whereas non-target-site resistance include detoxification, removal, reduced uptake or compensatory mechanisms (Table 1). Target-site mutations are most frequently reported (Lucas et al., 2015; Mair et al., 2016), with point mutations found in common fungicide targets like β-tubulin (Benzimidazole), CYP51A and B (DMI), CytB (Qol) and succinate dehydrogenase subunits SdhB, C and D (SDHI; Yin et al., 2023; Oliver et al., 2024), across several important fungal pathogens (Dorigan et al., 2023; Hawkins and Fraaije, 2021). Overexpression, copy number variation and heteroallelism of target genes can also confer fungicide resistance, as shown for the *CYP51* gene in *Blumeria graminis* f. sp. *tritici* (Arnold et al., 2024; Song et al., 2018; Stalder et al., 2023). Among non-target resistance mechanisms, detoxification often relies on more complicated pathways and is not commonly reported in fungal pathogens (Lucas et al., 2015). Removal often depends on over-expression of efflux transporter genes such as ATP binding cassette (ABC) and major facilitator superfamily (MFS) transporters, as observed in both clinical (Prasad and Rawal, 2014) and agricultural fungal pathogens (Cheng et al., 2023; Omrane et al., 2017). Although most cases involve single-nucleotide changes or gene duplications, additional mechanisms involving transposable elements and horizontal gene transfer also contribute to resistance evolution (Morogovsky et al., 2022; Omrane et al., 2017).

### 2.3 Resistance evolution

Resistance evolves when the variation across pathogen populations includes genetic traits that confer fitness advantages under selective pressure. When exposed to control measures, resistant genotypes survive and reproduce at higher frequency compared to susceptible genotypes (Lucas et al., 2015). The rate of resistance evolution depends on the amount of genetic variation present, the complexity and accessibility of the genetic change needed, and the strength of the selection pressure. In theory, larger populations tend to have more genetic variation (Papkou et al., 2021). This includes both locally dense populations and those connected by gene flow in open metapopulations (Treindl et al., 2023). Variation is also increased in organisms with high mutation rates (Bottery et al., 2024), those that undergo sexual recombination, and those that acquire genes through horizontal transfer (Barton, 2010; Taylor et al., 2017). These factors increase both the standing variation and the supply rate of new mutations, which together determine the adaptive potential of the population (Ament-Velásquez et al., 2022). Stronger selection pressure, such as repeated or high-dose applications of a single fungicide group will similarly lead to quick resistance evolution, provided there is sufficient genetic variation for the pathogen to adapt (Lucas et al., 2015). Notably, experiments with the cereal pathogen *Zymoseptoria tritici* showed that crossing sensitive and resistant isolates under fungicide pressure rapidly increased resistance allele frequencies (Kema et al., 2018). Pathogens exhibiting mixed reproductive strategies, such as multiple clonal generations combined with a single annual sexual cycle, pose the highest risk of overcoming host resistance and may similarly contribute to fungicide resistance (McDonald and Linde, 2002). To slow resistance evolution, it is critical to reduce both pathogen population size and the intensity of selection pressure. This involves limiting the advantage that resistant genotypes gain from control measures, for example, by rotating or combining treatments with different modes of action or using lower frequencies or lower doses. Such strategies help maintain susceptible genotypes in the population and thereby slow the spread of resistance.

The rate of resistance evolution also depends on pleiotropic effects and epistatic interactions of resistance-associated mutations. Resistance mutations can impose fitness costs, which may limit their spread in untreated environments (Hawkins and Fraaije, 2018). These costs arise from trade-offs, such as reduced enzyme efficiency or altered metabolic pathways, or resource allocation to protein overexpression or energy-dependent efflux. However, compensatory mutations can mitigate fitness costs, stabilizing resistance in populations (Schoustra et al., 2006). Understanding these dynamics is essential for designing effective management strategies (Corkley et al., 2022). For instance, allowing sufficient treatment gaps or rotating fungicides with different modes of action facilitates recovery of susceptible populations, thereby slowing down emergence of resistance.

### 2.4 Resistance risk, predictability and management

Assessing resistance risk involves evaluating the likelihood of resistance emergence and its potential impact (EPPO, 2015; Grimmer et al., 2015). The mode of action plays a crucial role, single-site fungicides are more prone to resistance than multi-site fungicides due to their specific target pathways, and overuse or misuse can accelerate this risk. Pathogens with high reproductive rates, large populations, and frequent recombination events also pose a greater threat. Proactive risk assessment helps guide fungicide use (Corkley et al., 2022). For example, laboratory mutagenesis studies and models can help to predict how resistance to a given compound might arise before it happens in the field, as well as highlighting the costs of resistance and compensating mutations (Hawkins and Fraaije, 2016). Resistance management strategies include the use of fungicide mixtures, which combine different modes of action, and/or alternations, which rotate fungicides. Integrated disease management strategies that combine chemical and non-chemical approaches help reduce reliance on any single method, thereby lowering selection pressure and slowing pathogen adaptation (Carolan et al., 2017). Optimizing dose rates and application timing is critical as higher doses can increase selection pressure, while lower doses may lead to weak control. Precision agriculture enables smarter pest management by enabling targeted preventative applications through improved disease forecasting tools, remote sensing and variable rate technology, which could enhance efficacy and reduce unnecessary fungicide use (Yang, 2020). Sustainable crop protection requires evolution-smart strategies integrating resistance breeding with a range of crop protection tools. It will need continuous monitoring, and innovation to address resistance challenges while ensuring long-term food security. Although these insights came too late to preserve the efficacy of historical fungicide classes, they offer valuable lessons to help prevent or delay resistance to newer fungicides and to emerging methods like RNAi-based crop protection.

## 3 Insights from fungal genomes

Fungi are amongst the most tractable of eukaryote genomes to sequence due to their limited size and frequent haploid state. There is a growing database of over 20,000 genomes belonging to more than 5000 species (NCBI accessed March 20, 2025). This growing genomic resource can provide major insights into how pathogens evolve resistance and adapt to new hosts. These data are instrumental for identifying promising genetic targets for future disease control strategies and for anticipating how resistance might emerge in response to novel technologies. In this section, we examine the different types of genetic changes that may serve either as potential targets for RNAi-based control or as mechanisms by which fungi could evolve resistance to such interventions.

### 3.1 Mutation

In species with large populations, standing diversity and many generations per year, beneficial single nucleotide mutants can emerge rapidly and fix in the population under strong selection pressure (Grimmer et al., 2015; Hawkins et al., 2019). Rust pathogens, in the basidiomycete order Pucciniales, include multiple examples of quickly adapting pathogens. New variants of stem rust (*Puccinia graminis*) have led to frequent breakdown of host immunity in modern wheat and barley varieties (Singh et al., 2015). Isolates of another cereal rust species, *P. striiformis* f. sp. *tritici*, were found to carry a DMI resistance-associated substitution in the *CYP51* gene or a substitution in *SdhC* (I85V) gene that is linked to SDHI resistance (Cook et al., 2021). Rapid emergence of resistance to multiple single-site fungicides (DMI, QoI and SDHI) was also observed for the soybean rust pathogen *Phakopsora pachyrhizi*, highlighting the speed of adaptation (Müller et al., 2021). The sexually reproducing wheat pathogen *Z. tritici* exhibits rapid adaption to key fungicide classes, with European populations consistently developing the first known resistance, corresponding to the intense fungicide use in the region (Feurtey et al., 2023).

### 3.2 Transposons

In many eukaryotes, including fungal plant pathogens, transposons are important drivers of genome evolution (Seidl and Thomma, 2017). In *Z. tritici*, transposon activity was shown to cause beneficial mutations for fungicide resistance (Oggenfuss et al., 2021). Transposon insertions upstream of a major facilitator gene (*MFS1*) or the promoter of transcription factor *Zymoseptoria* melanin regulation 1 (*Zmr1*) regulating melanin production conferred increased tolerance to stresses including fungicides in *Z. tritici* (Krishnan et al., 2018; Omrane et al., 2017). In *Monilinia fructicola*, the causative fungus of brown rot disease, a nested transposable element located upstream of the *CYP51* gene was linked to increased gene expression in resistant isolates, but was absent in highly-sensitive isolates (Durak and Özkilinç, 2025). Transposon activity was induced in *M. fructicola* following fungicide application (Chen et al., 2015), further accelerating the likelihood of adaptation. Transposon activation has also been observed during host plant infection, as part of a regulatory switch to activate pathogenicity genes (Fouché et al., 2020; Torres et al., 2021). In bacteria, SOS responses preserve DNA integrity at the cost of increased mutagenesis (Baharoglu and Mazel, 2014; Maslowska et al., 2019), and similar stress-induced mechanisms in fungal pathogens may promote hypermutator states under fungicide pressure or host defences.

There is growing evidence that mobile giant transposons can also accelerate evolution via horizontal gene transfer (Urquhart et al., 2024). In *Paecilomyces variotii* a giant transposon named *HEPHAESTUS* (~85 kb) was found to carry a gene cluster conferring metal resistance (Urquhart et al., 2022), suggesting it may play a role in mediating resistance to copper-based fungicides. Horizontal transfer of antifungal triazole resistance has been observed in experimental evolution studies with *Aspergillus fumigatus* (Morogovsky et al., 2022). Similar mechanisms could contribute to the future emergence of RNAi resistance, either by altering target genes or by disrupting dsRNA uptake pathways and components of the RNAi machinery.

### 3.3 Gain and loss of genome regions

The movement of plasmids carrying resistance genes is an important process in the evolution of antibiotic resistance in bacteria. Whilst this is not the case for fungicide resistance, recent research has shown that transposons, and the gain and loss of genomic regions, contribute to adaptive variation in fungi (Heckel, 2022; Oggenfuss et al., 2021; Tralamazza et al., 2024). Although variations in chromosome number or large-scale duplications often incur fitness costs (Todd et al., 2017), they can also generate genetic variation that supports adaptation, for example by enhancing virulence or conferring fungicide resistance (Ropars et al., 2018; Sionov et al., 2010). In *Erysiphe necator*, the grape powdery mildew pathogen, duplication of a 10 kb genomic region containing the *CYP51* gene has been associated with resistance to azole fungicide (Jones et al., 2014). Although gene loss can impact fungal fitness, as often seen with virulence factors such as effector genes, it has not typically been associated with fungicide resistance (Zaccaron and Stergiopoulos, 2024).

Fungal mini-chromosomes, which are small (<2 Mb), enriched in repetitive sequences, and carry few, mostly hypothetical genes, vary greatly among isolates and represent another genomic feature potentially involved in adaptation (Bertazzoni et al., 2018). Although mini-chromosomes have been occasionally linked to pathogenicity (Dijkstra et al., 2024; Ma et al., 2010; van Dam et al., 2017), their origin and functional roles remain largely elusive but their potential role in fungicide resistance warrants further investigation. In human fungal pathogens, whole chromosome duplications have been directly linked to increased virulence and drug resistance (Bing et al., 2020; Li et al., 2015). Collectively, genomic structural variants, including aneuploidy, dispensable chromosomes, and gene deletions or duplications, can accelerate fungal adaptation and may play a central role in the development of fungicide resistance under environmental stress (Hawkins et al., 2014; Steinhauer et al., 2019; Zaccaron and Stergiopoulos, 2024).

### 3.4 Epigenetic responses and small RNAs

Another poorly understood mechanism of resistance to antifungals involves RNA-mediated gene silencing (RNAi). For example, the lethal human pathogen *Mucor circinelloides* can develop transient resistance to antifungal drugs through an RNAi-based mechanism known as epimutation (Chang et al., 2019a). In this mechanism, small RNA (sRNA) molecules produced by the RNAi machinery of the pathogen trigger mRNA degradation to silence the gene targeted by the antifungal. These reversible epimutations are selected under drug pressure and can even be transmitted between sexual generations (Pérez-Arques et al., 2024), but sensitivity is typically restored over time in the absence of the drug. There is increasing evidence for a role of microRNAs (miRNAs) and micro-like RNAs (milRNAs) in pathogen-host interactions through gene silencing (Arslan and Ozkilinc, 2024; Gao et al., 2024). It is possible that these sRNAs along with their underlying machinery, may contribute to fungicide resistance or interfere with future RNAi-based control measures, particularly when targeting specific genes. Other epigenetic mechanisms, such as DNA methylation and histone modification, have also been implicated in fungicide resistance (Chang et al., 2019b).

## 4 Evolutionary principles for dsRNA-based control

The history of fungicide application and its pitfalls due to rapid resistance evolution as well as reduced societal acceptance contribute to the interest surrounding the use of alternative approaches such as RNAi-based crop protection (Zhao et al., 2024). In theory, dsRNA treatments can be designed using multiple target gene sequences selected from comprehensive genomic data, while minimizing off-target effects on non-target organisms. However, further research is needed to understand how insights from fungicide use and the increasing availability of fungal genomes can be leveraged to optimize dsRNA-based crop protection.

### 4.1 RNAi-based silencing

The dsRNAs applied in sprays are cleaved into small interfering RNAs (siRNAs) by either fungal or plant Dicer-like (DCL) proteins (Bernstein et al., 2001; Koch et al., 2016; Wang et al., 2016). Within fungal cells, the siRNAs bind to Argonaute (AGO) proteins in the cytoplasm, forming the RNA-induced silencing complex (RISC; Martinez et al. (2002)). This complex then specifically binds and degrades the complementary mRNA target, suppressing synthesis of the encoded protein. If the target is required for fungal growth or virulence, this can limit disease progression. Application of dsRNA has been successfully shown in various pathogens *in vitro*, including both foliar and soil-borne pathogens (Gu et al., 2019; Mosquera et al., 2025; Qiao et al., 2021), and in the field against *Botrytis cinerea* on chickpea and strawberry (Capriotti et al., 2024; Niño-Sánchez et al., 2022). Especially with the development of protective nano molecules, like BioClay™ (Mitter et al., 2017), stability and longevity of dsRNA applied on the plant are vastly improved, leading to improved protection in field conditions.

### 4.2 Balancing target specificity and durability

An ideal target for RNAi-based disease control would offer robust protection against a pathogen and with low risk of resistance development, while at the same time minimizing off-target effects. However, experience with conventional fungicides shows this is a delicate balance. Multi-site fungicides tend to have a lower risk of resistance development but higher risk of off-target toxicity, whereas single-site inhibitors are more precise but vulnerable to resistance through minor genetic changes, such as point mutations.

Fungicide target genes are often essential and highly conserved in fungal species, which enables broad spectrum activity across fungal pathogens. Modern fungicides are designed to bind with high specificity to their molecular targets, which generally minimizes off-target effects such as phytotoxicity or unintended impacts on non-target organisms, including humans. However, this specificity does not eliminate all concerns. Beneficial fungi, such as mycorrhizae, fungal endophytes (Poveda et al., 2022), and biocontrol agents like entomopathogenic (Karthi et al., 2024), or nematicidal fungi (Sánchez-Gómez et al., 2023), may still be adversely affected. Moreover, the high target specificity of these fungicides means that even minor evolutionary changes in the fungal target can significantly reduce their efficacy. Similarly, RNAi-based strategies that target essential fungal genes must carefully balance the risk of resistance development with the need to maintain a favourable safety profile. Even essential genes display some genetic variability in pathogen populations, which means specificity needs to be combined with sufficient sequence conservation. Growers often need to control multiple diseases in a single crop, so a highly sequence specific dsRNA would need to be used in combination either with other dsRNAs or with complementary control measures to be effective against other pathogens.

The degree of specificity of RNAi is crucial and one of the most important areas of research for this technology. Although effective gene silencing is generally thought to require complete sequence complementarity between sRNAs and their target transcripts, partial matches can still induce biological responses, which potentially leads to off-target effects (Huang et al., 2009; Neumeier and Meister, 2021; Zarrabian and Sherif, 2024). Furthermore, exactly how dsRNA is processed into a population of sRNAs and how this interacts with the organism’s native sRNA population, is still being investigated (Neumeier and Meister, 2021; Piombo et al., 2024).

In addition to sequence specificity, design features of dsRNA molecules significantly influence their silencing efficiency (Mosquera et al., 2025). These include targeting accessible regions of the mRNA, particularly exonic regions near the 3′-end, as well as optimizing the length of the dsRNA, which typically ranges from 150 to 550 nucleotides depending on the species. Other parameters, such as the GC content of gene, may also be important, as outlined by Mosquera et al. (2025). Advancing our understanding of how dsRNA is processed and what determines the specificity of silencing is essential. Only by moving beyond a trial-and-error approach can we fully leverage RNAi technology for precise and effective gene targeting.

A potential strategy to balance specificity and durability would be to use multiple site-specific dsRNAs targeting different genes, either within a single product or through rotational application. This approach could mitigate the risk of resistance development by requiring multiple independent mutations for successful adaptation, mimicking the principle behind mixing or rotation of conventional fungicides with different modes of action. To further reduce the likelihood of resistance emerging from a single mutation or gene duplication event, the targeted genes should be physically distant from one another. However, this strategy may also carry the risk of selecting for broader, more generalized tolerance (Ballu et al., 2023).

### 4.3 Potential targets

Each potential RNAi target involves specific trade-offs between efficacy, resistance risk, and environmental impact (Table 2). Most RNAi-based control efforts to date have focused on essential genes (Mosquera et al., 2025; Rosa et al., 2022), such as *CYP51* (Höfle et al., 2020; Koch et al., 2019), *β-tubulin* (Gu et al., 2019), chitin synthases (CHS; Saito et al., 2022), mitogen-activated protein kinases (MAPK; Degnan et al., 2023), translation elongation factor 1α (EF1-a; Degnan et al., 2023), and components of the RNAi machinery such as DCL and AGO (Qiao et al., 2021). Targeting these genes generally guarantees a higher and more stable efficiency in disease control since they are constitutively expressed and often occur as single copy. However, except for *AGO* and *DCL* genes (Werner et al., 2020), these genes have been subject to selection pressure from conventional fungicides, meaning that existing resistance mutations in pathogen populations must be carefully considered when designing dsRNA constructs.

Targeting essential genes imposes strong and continuous selection pressure during treatment on pathogen populations, a dynamic that has historically contributed to the rapid emergence and spread of resistance to chemical fungicides. As an alternative, RNAi strategies that modulate host-pathogen interactions, rather than directly killing the pathogen, may offer more durable and sustainable disease control. For instance, dsRNA constructs could target host susceptibility genes to reduce disease severity without exerting direct selective pressure on the pathogen (Zaidi et al., 2018). However, targeting host genes involved in pathogenicity-related processes presents its own set of challenges.

Ideally, the targeted genes should be constitutively or consistently expressed during pathogen colonization to ensure effective silencing regardless of when the dsRNA is applied. This is particularly important under field conditions, where aligning application timing with specific stages of pathogen infection is difficult. Additionally, any potential trade-offs, such as yield penalties due to altered host immunity or inadvertent silencing of genes in beneficial or non-target fungi, must be carefully considered and minimized.

Another promising strategy involves targeting fungal-specific stress-related pathway genes or species-specific pathogenicity factors. G-protein-coupled receptors (GPCRs), which allow fungi to sense and respond to environmental cues and thereby coordinate cellular functions related to survival, reproduction and virulence (Brown et al., 2018), are attractive candidates. Other potential targets include genes involved in appressorium formation, such as *PLS1* in *B. cinerea* (Spada et al., 2023), and *PG1* in *Magnaporthe grisea* (Soanes et al., 2002), or toxin production, such as *TRI5* in *Fusarium culmorum* (Tretiakova et al., 2022), *FUM1* in *F. verticillioides* (Johnson et al., 2018), and *BOT2* and *BOA6* in *B. cinerea* (Leisen et al., 2022). Silencing these genes would impair a pathogen’s ability to cause disease without necessarily eliminating the organism from the environment, unlike approaches that target core metabolic pathways and apply strong selection for survival. This strategy could limit resistance evolution especially for controlling opportunistic pathogens with broad host ranges.

Targeting host pathogenicity factors, including effector genes, can have important drawbacks. These genes are often located within fast-evolving, transposon-rich genomic regions (Dong et al., 2015; Torres et al., 2020), shaped by prolonged selection pressure from plant immune responses. In addition, only few effector genes are well-characterized and they tend to be highly variable among species. Many can evade plant resistance by rapidly evolving, or even by being lost entirely, particularly if they are redundant or lineage specific. However, RNAi approaches may still be suited for targeting more conserved, ‘core effectors’, those that are conserved within a species, functionally important and less likely to be lost without a fitness cost. These would potentially provide more durable targets despite the known variability associated with effectors.

### 4.4 dsRNA resistance evolution

For single-site fungicides, resistance commonly arises through target-site mutations that disrupt the binding of the chemical. Given that RNAi also relies on sequence-specific interactions between small interfering RNAs (siRNAs) and target mRNAs, similar resistance mechanisms could potentially occur through mutations that reduce siRNA–mRNA complementarity. However, unlike fungicides, where a single amino acid change at the binding site may be sufficient to confer resistance, RNAi may be affected by a broader range of mutations, including synonymous changes within the siRNA binding region. The threshold at which mismatches disrupt RNAi effectiveness remains poorly defined. Some degree of mismatch can be tolerated, but the impact likely depends on both the position of the mutation within the siRNA–mRNA duplex and the specific nucleotide change (McGeary et al., 2019). Importantly, longer dsRNA molecules generate multiple siRNAs targeting different regions of the same transcript, making it unlikely that a single mutation would completely abolish silencing. Instead, multiple independent mutations might be required to fully evade RNAi-mediated control. Further research is needed to better understand the mismatch tolerance of RNAi systems and the likelihood of resistance developing through target sequence variation.

RNAi-based crop protection holds great promise, but some fungal pathogens, such as *Z. tritici* (Kettles et al., 2019), *Trichoderma virens* and *Colletotrichum gloeosporioides* (Qiao et al., 2021), do not take up environmental dsRNA. The inability of some fungal pathogens to take up environmental dsRNA may be linked to differences in cell wall structure or composition, which could act as a physical or biochemical barrier which prevents dsRNA entry. The possibility arises that pathogens could evolve resistance by simply losing or downregulating the machinery for dsRNA uptake, a phenomenon already seen in laboratory settings. For instance, *Diabrotica virgifera* (western corn rootworm), under experimental evolution developed RNAi resistance by changing its gut physiology to limit dsRNA uptake (Khajuria et al., 2018). Similarly, point mutations in *DCL* genes have been shown to confer resistance to RNAi-based control of *F. asiaticum*, by impairing the accumulation of sRNAs (Gu et al., 2023). *Colletotrichum* species show low or no environmental dsRNA uptake, suggesting potential mechanisms that block uptake and confer RNAi resistance (Gu et al., 2019; Qiao et al., 2021). Several fungal species have independently evolved complete loss of RNAi pathways, including the plant pathogen *Ustilago maydis* (Laurie et al., 2008; Nicolas et al., 2013), suggesting that this may pose a risk as a potential resistance mechanism to RNAi-based control.

A key advantage of RNAi is the flexibility to adapt dsRNA sequences in response to the emergence of new pathogens and resistance emergence. This adaptability could allow for much faster responses than years typically required to breed and deploy new resistant crop varieties or the development and release of novel fungicides. Realising this potential, however, will rely on the development of a regulatory framework and safety evaluation systems that accommodate the rapid refinement of dsRNA-based pathogen control products.

## 5 Deployment strategies

The effective deployment of RNAi-based crop protection requires a carefully designed implementation that ensures efficacy, durability, regulatory compliance, and public acceptance. Application strategies should be designed with pathogen evolution in mind to reduce the emergence of resistance. Given that target-site mutations are the most reported resistance mechanism for chemical fungicides, RNAi strategies must proactively address similar risks.

### 5.1 Deployment

RNAi functions by modulating gene expression with high sequence specificity. Therefore, environmental factors, pathogen biology, and integration with existing pest management systems must also be considered when designing deployment strategies. Seasonal timing is critical, as RNAi application must coincide with the life cycle stage of the target pathogen when the target genes are highly expressed. For many pathogens, this means applying dsRNA treatments early in the infection process, before populations can reach damaging thresholds. Timing may be even more critical for RNAi than for fungicides, since dsRNA must be present and functional prior to the translation of the targeted gene, and dsRNA stability in field conditions is typically lower than that of chemical sprays. Environmental factors, including temperature (Darrington et al., 2025), UV exposure (San Miguel and Scott, 2016), humidity and rainfall (Mitter et al., 2017), can significantly influence RNA stability, uptake, and efficacy (Mosquera et al., 2025). For this reason, efforts to optimize dsRNA formulations, such as encapsulating dsRNA in nano-particles (Niño-Sánchez et al., 2022), to withstand these environmental challenges is essential to enhance persistence and improve consistency in challenging field environments.

### 5.2 5IPM

To ensure long-term durability, RNAi-based crop protection strategies should be embedded within a broader integrated pest management (IPM) framework rather than deployed as stand-alone solutions (Dara, 2019). IPM emphasizes the coordinated use of multiple control strategies to sustainably manage pest populations and delay resistance development. Within this context, RNAi-based crop protection can be combined with other complementary measures such as resistant crop varieties, crop rotation, biocontrol agents, biopesticides, beneficial microbes, and conventional agrochemicals. Additionally, stacking multiple dsRNA constructs that target different genes within the pest or host plant can further reduce the likelihood of resistance and enhance overall efficacy. This principle is already being applied in commercial systems. For instance, SmartStax® PRO corn integrates RNAi-based insect control with multiple *Bacillus thuringiensis* (Bt) toxins to provide distinct modes of action for pest suppression (Darlington et al., 2022). Such stacking substantially lowers the probability that a pest individual will simultaneously develop resistance to all control elements. Similar IPM-aligned strategies could be extended to RNAi-based foliar sprays or seed treatments. Nevertheless, additional research is essential to assess potential interactions among these tactics to avoid this risk of resistance evolution.

### 5.3 Regulatory framework

Another critical factor shaping the future of RNAi technologies in agriculture is the regulatory landscape. Although HIGS is more robust, as shown with SmartStax® PRO corn, current GMO regulations in especially the EU render this technology unfeasible. Currently, dsRNA-based products are subject to the same stringent approval processes as conventional chemical pesticides, often requiring three or more years to complete safety testing and registration. To date, only one RNAi spray product, *Calantha*, developed by GreenLight Biosciences for the control of Colorado potato beetles (Pallis et al., 2023), has reached the commercial market (Narva et al., 2025). While countries like the United States and Australia have introduced dedicated regulatory pathways for dsRNA-based products, Europe has yet to establish a comparable framework. In Europe dsRNA fungicides fall into the 1107/2009 law for conventional Plant Protection Products (PPP), for which the preliminary EFSA and European Commission (EC) approval and the subsequent molecule evaluation for risk assessment are required. However, RNAi-based fungicides act with completely different properties than conventional PPP and they should be assessed through specifically amended guidance protocols by the EC. This gap presents a significant barrier to innovation and timely adoption, stressing the urgent need for a novel regulatory protocol, one that not only addresses risk and safety assessment but also incorporates resistance monitoring from the outset, as is already required for conventional fungicides (EPPO, 2015). Notably, the Insecticide Resistance Action Committee (IRAC) has established a new mode of action category, Category 35: *RNA interference-mediated target suppressors*, for Ledprona, the active ingredient in Calantha. In addition to regulatory reform, broader adoption of RNAi will depend on effective communication with stakeholders and the public to raise awareness of the benefits of the technology and to address potential concerns. This might prevent societal backlashes as seen with the introduction of genetically modified organisms (GMOs). Early engagement with regulatory agencies during the development process will also be crucial. Such collaboration can help align expectations, ensure compliance, and prevent delays in market entry, ultimately facilitating the responsible deployment of RNAi technologies in sustainable pest and disease management.

## 6 Conclusions

Fungal plant pathogens remain a major threat to global food security. While traditional fungicides have significantly reduced crop losses over the past century, their widespread use has come with substantial environmental costs and the emergence of fungicide resistance. The advent of genomic technologies has transformed our understanding of fungal biology, revealing remarkable genome plasticity and variability. In addition to point mutations, mechanisms such as transposon activity, horizontal gene transfer, dispensable chromosomes, and epigenetic modifications are now recognized as key drivers of fungal adaptation.

Technologies based on dsRNA offer a promising and highly targeted approach to fungal disease control. However, while targeting essential genes can provide strong protective effects, it also exerts intense selection pressure, which may accelerate the evolution of resistance as observed with conventional fungicides. Similarly, targeting genes within the RNAi machinery poses the risk of selecting for mutants that lose the machinery entirely, potentially rendering the technology inviable. Therefore, ideal gene targets should be carefully chosen to balance reducing off-target effects and limiting the risk of resistance development. Considerations include whether target genes should be essential for basic survival or pathogenesis-related; conserved or lineage-specific; and located in conserved core genomic regions.

Several important scientific questions must still be addressed before the full potential of RNAi-based pathogen control can be realized. A deeper understanding of the natural roles of RNAi and small (s)RNAs in fungi is needed, especially regarding how exogenous dsRNAs are processed and how they interact with endogenous RNA pathways. It also remains unclear why some fungal species efficiently absorb environmental dsRNA while others do not. This difference is likely influenced by factors beyond the simple presence or absence of uptake machinery. Elucidating the reasons behind the non-uptake of dsRNA in some pathogens could inform the development of more effective delivery methods and expand the range of susceptible pathogens. How these traits relate to fungal fitness and pathogenicity will determine the risk of pan-RNAi resistance evolving through the loss of dsRNA uptake in previously susceptible species.

Resistance evolution is an inherent risk for any control strategy that imposes strong selective pressure. Laboratory-based selection experiments, as used in fungicide and antimicrobial resistance research, will be vital for anticipating resistance development in response to dsRNA treatments. These studies can support resistance risk assessments and help guide sustainable deployment strategies. Even if RNAi successfully controls a target pathogen, ecological niches may quickly be filled by other pathogenic species, including those that naturally lack RNAi pathways and may possess innate resistance traits. For this reason, RNAi technologies should be embedded within IPM frameworks that combine multiple complementary strategies to ensure long-term effectiveness.

It is equally important to ensure that the development and deployment of dsRNA technologies is accompanied by clear and transparent communication with the public and relevant stakeholders. Past experiences with GMOs have shown that lack of engagement can lead to societal objections. Open dialogue about the benefits, limitations, and safety of RNAi-based tools will be critical for building public trust, informing policy, and promoting responsible adoption.

In conclusion, external dsRNA application represents a powerful and potentially sustainable tool for managing fungal crop diseases. With focused research, strategic implementation, and open communication, RNAi can become a valuable addition to the integrated crop protection toolbox.

## Figures and Tables

**Table 1:** Overview of resistance mechanisms and risks.

Resistance type	Mechanism	RNAi resistance risk	Unknown factors
Target site mutations	Point mutations alter the target protein’s binding site, reducing fungicide binding	Corresponding mRNA changes may reduce siRNA binding and RNAi effectiveness	Number, position, and type of mismatches in siRNA needed to cause RNAi failure; effects of dsRNA length and GC content
Target-site overexpression	Increased expression of target gene via promoter or transcription factor changes	Higher gene expression may require higher dsRNA doses to maintain control	Whether overexpression can be overcome with applying specific dsRNA doses
Non-target-site resistance	Enhanced fungicide efflux, detoxification and metabolic bypass mechanisms	Some pathogens lack RNAi susceptibility, often due to RNA uptake issues; could cause broad RNAi resistance	Mechanisms underlying RNAi non-susceptibility across species; potential fitness costs or loss of pathogenicity if such mechanisms evolve in susceptible fungi

**Table 2:** RNAi targets and trade-offs.

RNAi Target Type	Advantages	Disadvantages
Essential/housekeeping genes^a^	Stops pathogen growth; adaptable to multiple pathogens	Strong continuous selection for resistance; risk to non-target species
Pathogenicity-related genes	Stops infection; low off-target effects	High resistance risk due to gene evolvability under host pressure; sensitive to timing of gene expression
Conserved genes^a^	Broad-spectrum activity; lower resistance risk if functionally constrained	Possible off-target effects in non-pest species
Lineage-specific genes	Minimizes off-target effects	Higher resistance risk since genes may be dispensable
Constitutively expressed genes	Flexible timing; potential curative effects	Continuous selection increases resistance risk
Stage-specific genes	Reduced resistance selection due to limited expression period	Precise timing required; only protective if expressed early
Single target	Simple design and development	High resistance risk from single-gene mutations
Stacks/mixtures targeting multiple genes	Lower target-site resistance risk; may improve efficacy	Potential for non-target-site resistance and pan-RNAi resistance
Combining RNAi with other controls	Reduces resistance risk; broader activity; more robust control	Higher cost; formulation/timing challenges; may not align with replacement expectations

a Essential/housekeeping genes are functional definitions, while conserved is an evolutionarily definition, though often overlapping, some non-essential genes like effectors could be conserved.
